# Cuffless Blood Pressure Estimation Based on Monte Carlo Simulation Using Photoplethysmography Signals

**DOI:** 10.3390/s22031175

**Published:** 2022-02-04

**Authors:** Chowdhury Azimul Haque, Tae-Ho Kwon, Ki-Doo Kim

**Affiliations:** Department of Electronics Engineering, Kookmin University, Seoul 02707, Korea; c_azimul@kookmin.ac.kr (C.A.H.); kmjkth@kookmin.ac.kr (T.-H.K.)

**Keywords:** blood pressure, cuffless, Monte Carlo simulation, photoplethysmography, machine learning

## Abstract

Blood pressure measurements are one of the most routinely performed medical tests globally. Blood pressure is an important metric since it provides information that can be used to diagnose several vascular diseases. Conventional blood pressure measurement systems use cuff-based devices to measure the blood pressure, which may be uncomfortable and sometimes burdensome to the subjects. Therefore, in this study, we propose a cuffless blood pressure estimation model based on Monte Carlo simulation (MCS). We propose a heterogeneous finger model for the MCS at wavelengths of 905 nm and 940 nm. After recording the photon intensities from the MCS over a certain range of blood pressure values, the actual photoplethysmography (PPG) signals were used to estimate blood pressure. We used both publicly available and self-made datasets to evaluate the performance of the proposed model. In case of the publicly available dataset for transmission-type MCS, the mean absolute errors are 3.32 ± 6.03 mmHg for systolic blood pressure (SBP), 2.02 ± 2.64 mmHg for diastolic blood pressure (DBP), and 1.76 ± 2.8 mmHg for mean arterial pressure (MAP). The self-made dataset is used for both transmission- and reflection-type MCSs; its mean absolute errors are 2.54 ± 4.24 mmHg for SBP, 1.49 ± 2.82 mmHg for DBP, and 1.51 ± 2.41 mmHg for MAP in the transmission-type case as well as 3.35 ± 5.06 mmHg for SBP, 2.07 ± 2.83 mmHg for DBP, and 2.12 ± 2.83 mmHg for MAP in the reflection-type case. The estimated results of the SBP and DBP satisfy the requirements of the Association for the Advancement of Medical Instrumentation (AAMI) standards and are within Grade A according to the British Hypertension Society (BHS) standards. These results show that the proposed model is efficient for estimating blood pressures using fingertip PPG signals.

## 1. Introduction

The pressure of the circulating blood against the walls of the arteries is called blood pressure (BP). The heart pumps blood through the circulatory system, which is the primary cause of the pressure. BP, along with the respiratory rate, heart rate, oxygen saturation, and body temperature, constitute the vital signs that medical doctors use to assess patient health. Therefore, monitoring BP for maintenance within the normal range is very important for planning a healthy life. BP is generally expressed as a fraction, where the numerator represents the systolic blood pressure (SBP) and the denominator represents the diastolic blood pressure (DBP). According to the American Heart Association [1], BP less than 120/80 mmHg is considered normal. The BP ranges for various health conditions are shown in Table 1.

Photoplethysmography (PPG) is an optical method of detecting changes in the blood volume in the microvascular layer of tissues. PPG is frequently used for noninvasive measurements from the skin surface. PPG sensing is a low-cost, easy-to-use technology that requires only a few electronic components for acquiring signals. A light source is used to illuminate the skin surface, and a detector is used to detect the light from the source. Depending on the placement of the light source and detector, PPG signals can be divided into two types: transmission and reflection [2]. If the light source and detector are placed on the same side of the measured area, it is considered as reflection-type PPG, and if they are placed on opposite sides, it is considered as transmission-type PPG.

Over the last few years, PPG has been used to monitor and evaluate various physiological parameters, such as blood glucose, heart rate variability, BP, arterial oxygen saturation (SpO_2_), and glycated hemoglobin (HbA1c) [3,4,5,6,7]. In [3], the authors developed a gray-box model to estimate HbA1c using a digital pulse waveform. Alqaraawi et al. [4] estimated heart rate variability with PPG signals using a Bayesian learning algorithm. The authors of [5] developed a machine-learning-based model to estimate blood glucose levels from PPG signals; they reported an all-purpose system that collects PPG signals to estimate blood glucose levels with two machine-learning models: namely, random forest and XGBoost. BP measurement is a popular application of PPG signals, and several studies have reported various algorithms and models for estimating BP using PPG signals. Mousavi et al. [6] described an algorithm for estimating BP using a whole-based method, which is a feature formation technique in which the time-domain features from PPG and ECG or only from PPG are extracted at specific intervals; they also reported a method of distinguishing between appropriate and inappropriate PPG signals to estimate BP. Machine-learning and deep-learning techniques have also been used for BP measurements. The authors of [7] extracted time- and frequency-domain features from PPG signals and used them to estimate BP with a machine-learning approach; they also used a feature selection algorithm to reduce the computational complexity of the model, and their study reported that the Gaussian process regression (GPR) performs best for estimating SBP and DBP. Athaya et al. [8] proposed a method of estimating arterial blood pressure (ABP) waveforms using PPG for continuous BP measurements. In [8], a U-net architecture-based approach was described to estimate the ABP waveforms.

Monte Carlo simulation (MCS) is an adaptive computational technique that uses a random sampling procedure from a probability distribution; it is a widely used technique for simulating photon propagations in biological tissues. The basic principle of MCS in tissue results in a random walk process for transmitting light through biological tissues [9]. Each photon packet in the simulation has an initial weight, which is assumed to be 1. Then, the absorption and scattering coefficients of the tissue model are used to illustrate the probability of absorption and scattering for a unit path length [10]. The anisotropy factor g, which is the normal cosine of the scattering angle, determines the probability distribution of the scattering angle. The change in the refractive index n determines the angle of refraction, which changes between two regions of the tissue model or at the air–tissue interface. The fraction of the photon packet leaving on the same side of the medium is recorded as the reflected photon intensity (weight), and the fraction leaving on the opposite side of the medium is considered as the transmitted photon intensity [11]. Photon transport in biological tissues has become one of the popular methods in biomedical applications for estimating health parameters, such as BP and blood glucose concentration. MCS for light propagation in tissues is an important tool for understanding how light interacts with biological tissues [12], and this understanding can be used to noninvasively estimate the health parameters.

Cuffless measurement of BP is a new research trend that reduces the drawbacks of conventional BP monitoring. The conventional BP measurements have several problems, such as invasive monitoring and cuff-based or manual operating procedures. Kachuee et al. [13] proposed a cuffless BP estimation algorithm based on the pulse arrival times. According to the British Hypertension Society (BHS) standard, the results of their estimations achieve grade A for DBP and grade B for the mean arterial pressure (MAP). The authors of [14] developed a hybrid deep-learning-based model to predict BP from PPG and ECG signals. They implemented an automatic feature extraction layer in a deep-learning model to extract the optimal features from PPG signals for predicting BP. Atomi et al. [15] designed a BP estimation model using wrist-type PPG sensor signals and machine-learning models; they also developed a cloud system to store the medical information of patients and provide them appropriate advice. Esmaelpoor et al. [16] proposed a multistage deep-neural-network-based model for estimating the SBP and DBP; their model consists of two successive stages, where the first stage involves two convolutional neural networks to extract features from PPG signals and the second stage captures temporal dependencies using long short-term memory (LSTM). Then, their model incorporates these two stages and estimates the SBP and DBP. A real-time BP measurement algorithm was proposed in [17], whose random forest model outperforms all other machine-learning models based on feature vectors. However, machine-learning or deep-learning-based models for estimating BP are generally very complex and rely mostly on the signal measurement sites.

In our study, we propose the MCS-based model for estimating BP using fingertip PPG signals. We executed the MCS for both transmission and reflection types with wavelengths of 905 nm and 940 nm. For the MCS, we propose a finger model with the bio-optical properties of the layers for wavelengths of 905 nm and 940 nm. To evaluate the proposed model, we consider two datasets: a publicly available dataset and a dataset created by us. The publicly available dataset was used to evaluate the transmission-type MCS at 905 nm, and our self-made dataset was used for both transmission- and reflection-type MCSs at 940 nm.

The remainder of this study is organized as follows. Section 2 describes the materials and methods of this study, including the proposed finger description, MCS procedure, dataset description, and calibration model. Section 3 describes the model evaluation results. Finally, Section 4 present the conclusions of the study.

## 2. Materials and Methods

**Figure 1:**
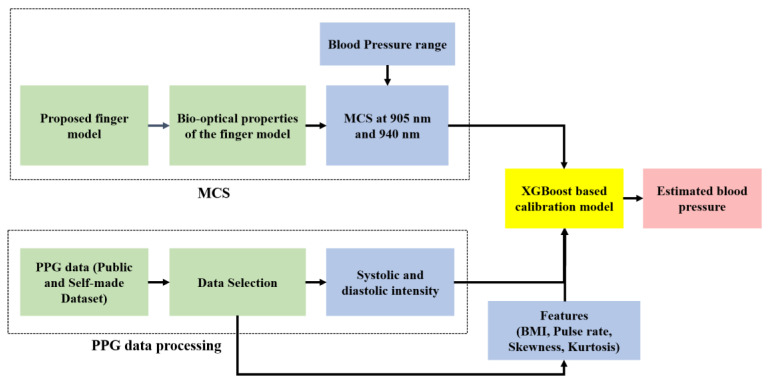
Workflow diagram of the proposed model.

The workflow diagram of the proposed model is shown in Figure 1, and the process is described sequentially.

For blood pressure estimation, a Monte Carlo simulation (MCS) is executed in the proposed finger model at two wavelengths: 905 nm and 940 nm. In this study, we proposed a finger model that uses MCS to estimate blood pressure. As a result, we needed wavelengths that penetrate deeper into the skin to produce more precise MCS intensities. For this reason, the infrared wavelength was chosen. The penetration depth of a wavelength in biological tissue depends on the scattering and absorption coefficients of the skin for that wavelength. If a wavelength of light scatters less in the tissue, it can penetrate deeper into the skin. The explanation for this is that scattering causes light dispersion in the tissue, which decreases the energy density with increasing depth. As a result, IR wavelengths penetrate deeper into the skin than other wavelengths [18,19].

### 2.1. Proposed Model

**Figure 2:**
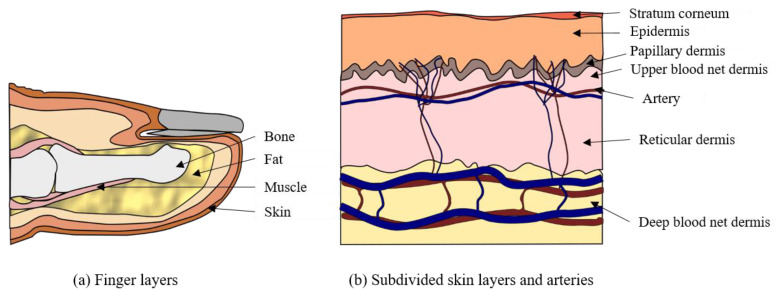
Proposed finger model.

A heterogeneous finger model was considered for photon transport in the MCS. In general, the spatial distribution of blood and chromophores in a finger varies with depth, but the anatomical constituents can be considered as constants. This approximation allows the finger to be considered a multilayered tissue. Then, the proposed finger model is divided into four main layers: skin, muscle, fat, and bone. The skin is subdivided into six dermal layers: stratum corneum, epidermis, papillary dermis, upper blood net dermis, reticular dermis, and deep blood net dermis. To measure BP, we considered that the artery exists in the dermal layer. Figure 2a shows the layers of the proposed finger model, and Figure 2b shows the subdivided skin layers and arteries present therein.

Table 2 shows the thickness of the finger layers and subdivided skin layers. Since the diameter of the artery is considered to vary according to the BP, the corresponding changes will be discussed in Section 2.2.2.

### 2.2. Bio-Optical Properties

#### 2.2.1. Properties of the Proposed Finger Layers

The bio-optical properties of the layers of the proposed finger model are required for the MCS to record the photon intensities. After entering the tissue medium, the photons are absorbed by the medium, scattered from the surface, or scattered within the medium, so the bio-optical properties are required for the MCS of our proposed finger model. The absorption coefficient $\;\mu_{a}$, scattering coefficient$\;\mu_{s}$, anisotropy factor *g*, and refractive index *n* are considered for the bio-optical properties of tissues. The general equation of the absorption coefficient $\mu_{a}$ for a heterogeneous biological tissue is represented by Equation (1).

(1) $$\mu_{a}\left(\lambda \right)=\sum_{i=1}^{k}\left(\mu_{a_{i}}\left(\lambda \right)\times V_{i}\right)+\mu_{a}^{\left(0\right)}\left(\lambda \right)\times \left(1-\sum_{i=1}^{k}V_{i}\right)\;$$

where $V_{i}$ is the volume fraction of the *i*-th layer, and *k* is the total number of layers; $\mu_{a}^{\left(0\right)}\left(\lambda \right)$ is the baseline absorption coefficient and is expressed as in Equation (2).

(2) $$\mu_{a}^{\left(0\right)}\left(\lambda \right)=7.84\times 10^{7}\times \lambda^{-3.255}$$

Only melanin and water were considered to calculate the absorption coefficients of the stratum corneum and epidermis because these layers do not contain blood cells. The absorption coefficient of the epidermis is expressed as follows [22]:

(3) $$\mu_{a}^{\left(Epi\right)}\left(\lambda \right)=(V_{m}\times \mu_{a_{m}}\left(\lambda \right))+\left(V_{w}\times \mu_{a_{wat}}\left(\lambda \right)\right)+\left[1-\left(V_{m}+V_{w}\right)\right]\times \mu_{a}^{\left(0\right)}\left(\lambda \right)$$

(4) $$\mu_{a_{m}}\left(\lambda \right)=6.6\times 10^{10}\times \lambda^{-3.3}$$

where $V_{m}$ is the melanin volume fraction, which is considered as 10%, $\mu_{a_{m}}\left(\lambda \right)$ is the absorption coefficient of melanin at wavelength λ, $V_{w}$ is the water volume fraction in the epidermis, and $\mu_{a_{wat}}\left(\lambda \right)$ is the absorption coefficient of water at wavelength λ.

The equation for calculating the absorption coefficient of the stratum corneum is adopted from [23] and is expressed as follows.

(5) $$\mu_{a}^{\left(StC\right)}\left(\lambda \right)=\left[\left(0.1-0.3\times 10^{-4}\lambda \right)+0.125\times \mu_{a}^{\left(0\right)}\left(\lambda \right)\right]\times \left(1-V_{w}\right)+V_{w}\mu_{a_{Wat}}\left(\lambda \right)$$

**Figure 3:**
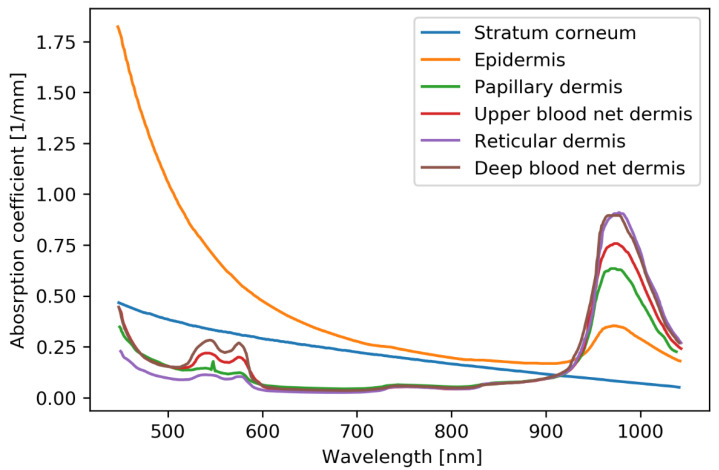
Absorption coefficients of the subdermal layers [23].

In the proposed finger model, we consider a total of nine layers, including the subdivided dermal layers. The absorption coefficient $\mu_{a}$ and scattering coefficient $\;\mu_{s}$ of the subdivided dermal layers are adopted from the data in [23] for wavelengths ranging from 400 to 1100 nm, and the absorption coefficient values are shown in Figure 3. The blood and water volume fractions used for calculating the absorption coefficients in [23] are listed in Table 3.

The bio-optical properties of the other finger layers, such as fat, muscle, and bone, are adopted from [10,20,21]. We have used the bio-optical properties of the proposed finger model for wavelengths of 905 nm and 940 nm. Table 4 lists the bio-optical properties used in this study.

#### 2.2.2. Artery Properties

In the proposed finger model, we consider that the artery is located between the upper and deep blood net dermis layers. Owing to changes in the ABP, the diameter of the artery will change [24], and the total volume of the artery will also change. Since the absorption coefficient of the artery is related to the volume, the changes in the arterial diameter are important for recording photon intensities to estimate BP by MCS. The absorption coefficient of the artery at wavelength  λ can be expressed as in Equation (6).

(6) $$\mu_{a}^{art}\left(\lambda \right)=v_{blood}\times \mu_{blood}\left(\lambda \right)+v_{vw\;}\times \mu_{vw}\left(\lambda \right)$$

where $v_{blood}$ and $v_{vw}$ denote the volume fractions of the whole blood in the artery and vessel wall, respectively; $\mu_{blood}$ and $\mu_{vw}$ denote the absorption coefficients of the whole blood and vessel wall, respectively.

The volume fractions of whole blood in the artery and vessel wall depend on the arterial volume; thus, $v_{blood}$ and $v_{vw}$ can be expressed as Equations (7) and (8), respectively.

(7) $$v_{blood}=\frac{V_{B}}{V_{T}}$$

(8) $$v_{vw}=\frac{V_{VW}}{V_{T}}$$

where $V_{B}$, $V_{VW}$, and $V_{T}$ denote the volumes of blood in the artery, vessel wall, and total artery, respectively. To perform the MCS in this study, we considered the artery as a cylinder and calculated its volume. To calculate the volume of the vessel wall, the thickness of the artery is considered as 0.2 mm [25]. As mentioned above, the arterial diameter changes according to the change in arterial pressure; the pressure–diameter relation was derived through curve fitting of the data in [24]. The pressure–diameter relation can be expressed as Equation (9).

(9) $$d=0.8531\times p^{0.1023}$$

**Figure 4:**
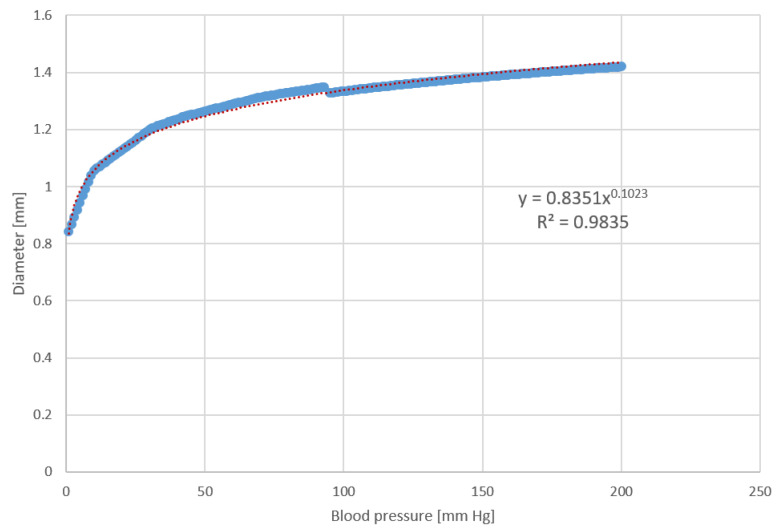
Pressure–diameter relation of the artery.

In Equation (9), *d* denotes the arterial diameter and *p* denotes the arterial pressure. The fitted curve and coefficient of determination (R^2^) value for the curve are shown in Figure 4.

Absorption coefficients of the whole blood and artery vessel wall at wavelengths of 905 nm and 940 nm are adopted from [26,27] and listed in Table 5.

### 2.3. Monte Carlo Simulation (MCS)

In this study, we present the MCS-based model for estimating BP (SBP, DBP) without using a cuff. The MCS was chosen for photon propagation in the proposed biological tissue owing to its flexibility. The proposed heterogeneous finger model contains four basic layers: skin, muscle, fat, and bone. The skin is subdivided into six dermal layers for the MCS to estimate the BP more accurately. In this study, MCS was performed for both the transmission and reflection configurations. The transmission wavelengths are 905 nm and 940 nm, and the reflection wavelength is 940 nm. In the reflection-type scheme, the distance between the light source and detector is 0.4 mm, whereas in the transmission-type scheme, the detector is 10.06 mm away from the opposite side of the model.

**Figure 5:**
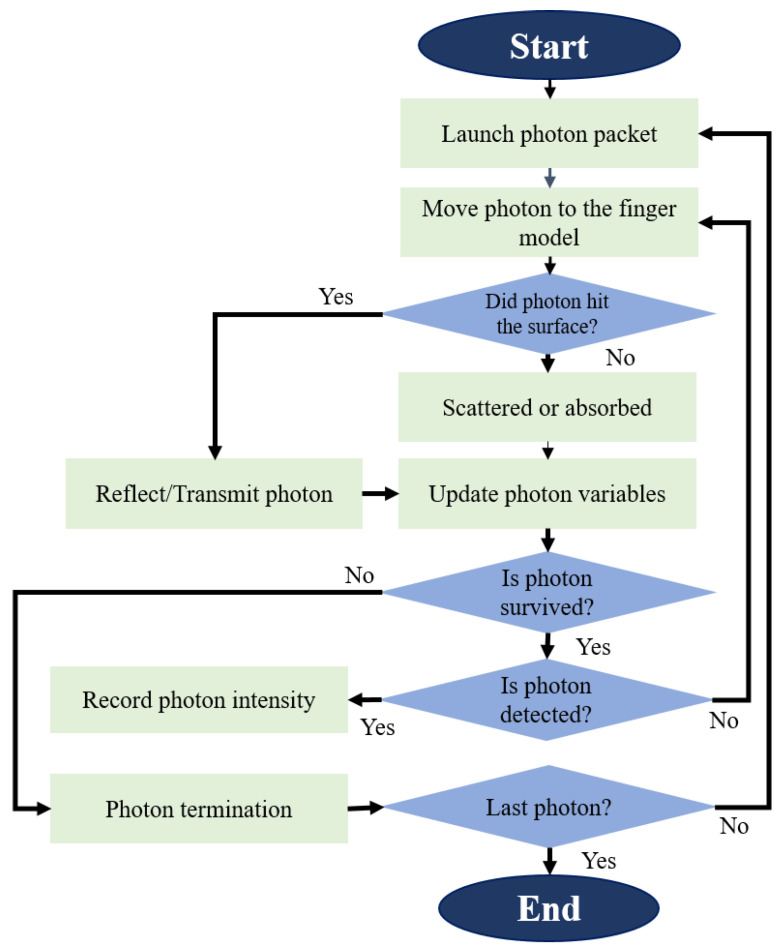
Flowchart of the MCS in this study.

To propagate photons in the proposed finger model, we considered the voxel-based Monte Carlo algorithm described in [28], where 3D multilayer volumes were designed to assign a single integer to each voxel to indicate the index of the layer. The photon packet is randomly scattered or absorbed by the layers once it enters the finger model. Then, the BP is determined by collecting the photons that reach the detector and evaluating their intensities. Figure 5 shows the flow chart of the MCS used in this study.

Initially, a photon packet was released into the finger model with a weight of 1 (one). The step size (*l*) was estimated by random sampling of the photon scattering probability [29]. When a photon hits the surface of the proposed model, it is reflected or transmitted. The photon variables (position vector, direction vector, and weight) are updated after each reflection or transmission. The cosine of the scattering angle can be defined by the Henyey–Greenstein phase function [30] and is expressed by Equation (10):

(10) $$cos\theta =\{\begin{array}{c} \frac{1}{2g}\left[1+g^{2}-{\left(\frac{1-g^{2}}{1-g+2g\xi }\right)}^{2}\right]if\;g\neq 0\\ 1-2\xi \;if\;g=0 \end{array}\;$$

where g denotes the scattering anisotropy; g=0 indicates isotropic scattering; and g=1  indicates forward-directed scattering. The step size (*l*) is determined by the random number ξ (0 < ξ < 1), absorption coefficient ($\mu_{a})$, and scattering coefficient ($\mu_{s}$), and is expressed as Equation (11).

(11) $$l=-\frac{ln\xi }{\mu_{a}+\mu_{s}}$$

### 2.4. Blood Pressure Range Selection for MCS

In Section 2.2.2, the change in the arterial diameter due to pressure is described. The bio-optical properties of the artery will change according to Equation (6). To perform the MCS with the proposed model, the BP range was chosen by taking into account the surge and dipping of the SBP and DBP. The maximum surge of 45 mmHg of BP was observed in the morning [31], and maximum dipping was observed when the DBP was 25.5% of the normal BP [32]. We chose a BP range between 42 and 200 mmHg for the MCS of the proposed finger model by taking into account the surge and dipping in BP. The increment of BP was set to 1 mmHg.

### 2.5. Data Selection and Acquisition

Since wavelengths that penetrate deep into the skin were required to generate accurate photon intensities, infrared wavelengths were chosen in this study. Two PPG datasets were used to evaluate the performances of the proposed model in both transmission and reflection modes. For the transmission type, we used a publicly available PPG dataset [33]. We collected PPG signals from 30 volunteers according to the IRB Protocol of Kookmin University [protocol number: KMU-202006-HR-237], along with the SBP and DBP, to evaluate the model for both transmission and reflection type. To better represent this work, the publicly available dataset from 100 subjects is referred to as the public dataset, and that created by us is referred to as the self-made dataset. The data demographics and data acquisition protocol are as follows.

#### 2.5.1. Public Dataset

A public dataset was considered to evaluate the transmission-type MCS for estimating BP. This dataset integrates 657 segments of PPG signals and BP (SBP and DBP) data from 219 subjects; that is, there are three segments of PPG signals for each subject. Of the 219 subjects, 104 were male and 115 were female. The PPG signals are recorded at a wavelength of 905 nm at a 1 kHz sampling rate for 2.1 s. The SBP and DBP were measured using an Omron HEM 7201 upper arm blood pressure monitor. The accuracy validation of this device can be found in [34]. Table 6 shows the statistical information of the dataset.

Of the 657 PPG signal segments from 219 subjects, the signals of 100 subjects (300 segments) were selected based on signal quality, which is evaluated by calculating the skewness of the signal. The results of [35] and [36] showed that the skewness and kurtosis of a signal performed better than the other features, such as perfusion index, zero-crossing rate, and entropy, when evaluating signal quality. After calculating the skewness and kurtosis of each segment, the sum of the skewness values for each subject segment was calculated. The PPG signals (based on the sum of skewness) from the top 100 subjects were selected for the study. Table 7 lists the statistical information for the selected 100 subjects.

#### 2.5.2. Self-Made Dataset

To estimate BP by MCS, 30 subjects voluntarily agreed to provide PPG signals along with their SBP and DBP information. Written consent was obtained when the volunteers agreed to provide data for this study according to the Kookmin University IRB protocol. Of the 30 subjects, 16 were male and 14 were female (refer to Table S2 of the Supplementary Document for detailed information). From each subject, 240 s of PPG signals were recorded with a sampling rate of 83 Hz. Statistical information regarding the subjects is listed in Table 8.

Along with the PPG signals of the subjects, BP and pulse rate values were collected. The BP was measured using an Omron HEM-7130 upper arm blood pressure measurement device [37]. The system accuracy results for the Omron HEM-7130 is ±3 mmHg. To eliminate external influences, such as movement and electromagnetic disturbances, the subjects were instructed to remain stable during the PPG signal recordings. For stress-free BP measurements, the subjects were asked to not consume caffeinated beverages 1 h before the measurements and data collection. Since the subjects were in a stable position and no other sudden movements or clinical situations occurred, the possibility of blood pressure change during the PPG signal acquisition period is very small. Moreover, since the blood pressure measurement devices are based on the oscillometric method, which is independent of temperate and atmospheric pressure [38], the variations of environmental conditions are not considered.

### 2.6. Data Processing and Calibration

**Figure 6:**
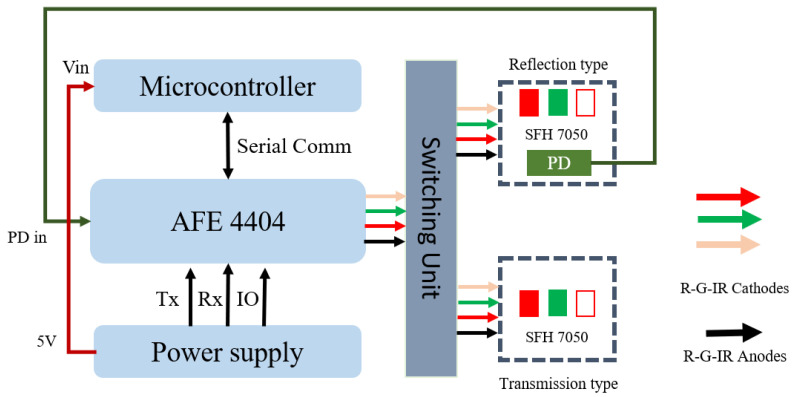
Block diagram of proposed hardware for data acquisition.

The data table for photon intensities was obtained after MCS with the set BP range (42–200 mmHg). Photon intensities for two wavelengths (905 nm and 940 nm) were observed for the BP range, and the intensity data table has 158 data points for both wavelengths. A hardware system was proposed for collecting the PPG signals from the subjects. The ESP32-PICO-V3 [39] was used to control the entire system as a processing unit. This microcontroller includes an on-chip radio-frequency communication system, allowing data to be sent to a remote server without the use of an external communication module. The reflection- and transmission-type PPG signals were collected using a surface-mounted device (SMD) module (SFH 7050) [40]; this module includes a photodetector (PD) and three different light-emitting diodes (LEDs): green (530 nm), red (660 nm), and infrared or IR (940 nm). The photodetector has a sensitivity range of 400 to 1100 nm. The LEDs and PD were controlled by a bio-sensing analog front end (AFE; AFE 4404) [41]. The three LEDs (green, red, and IR) were assigned to AFE 4404’s three transmitter pins (Tx1, Tx2, and Tx3). The next step was choosing between reflection- and transmission-type PPG signal acquisition. When the reflection type is activated, the LEDs on the same side of the PD light up. During operation in the transmission type, the LEDs on the opposite side of the PD are illuminated. A solid-state relay (optocoupler MOSFET, CPC2030N) was used to select the type. A two-input NAND gate (74AUP2G00DC-Q100H) is used to prevent both types (reflection and transmission) from being operated at the same time. The optocoupler activates the type based on the output of the NAND gate. Figure 6 illustrates the block diagram of the proposed hardware for data acquisition.

**Figure 7:**
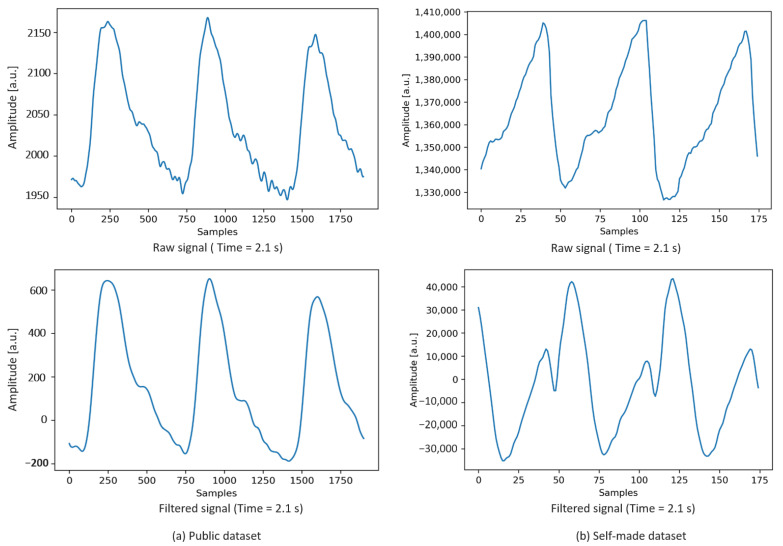
PPG signal examples.

In this study, the PPG signal of the IR wavelength was considered. Therefore, we collected PPG data with 22-bit resolution and stored it in the range of 425,000 for the IR wavelength. Then, we calibrated the PPG data with MCS samples after collection from the subjects. To estimate the SBP and DBP, the peak detection algorithm described in [42] was used to detect the systolic peak intensities of the PPG signals. The pulse onset, the starting point of one pulse of the PPG signal, is detected for estimating the DBP. To detect the pulse onset in the PPG signal, we first invert the PPG signal and then implement the peak detection algorithm to detect the peak. The detected peak of the inverse signal is referred to the pulse onset of the PPG signal. In this study, the pulse onset intensity of the PPG signal is considered as the diastolic intensity and used to estimate the DBP. According to the peak detection algorithm, the baseline wander has been eliminated by using a bandpass filter. The PPG signals from the dataset [33] were filtered using a 6th-order bandpass Butterworth filter with a cutoff frequency of 0.5–25 Hz. Among the other filtering processes, this filter provides a more accurate signal for this dataset [7]. For our self-made dataset, to remove baseline wander and high-frequency noise, a 6th-order bandpass Butterworth filter with a cutoff frequency of 0.5–8 Hz was used. Both high-frequency noises and baseline wandering due to respiratory factors are eliminated by using the bandpass filter on the PPG signals of the datasets. Figure 7 shows the raw and filtered signal example of both public and self-made datasets. Figure 7a illustrates the signal example for the public dataset, and Figure 7b shows the signal example for the self-made dataset.

**Figure 8:**
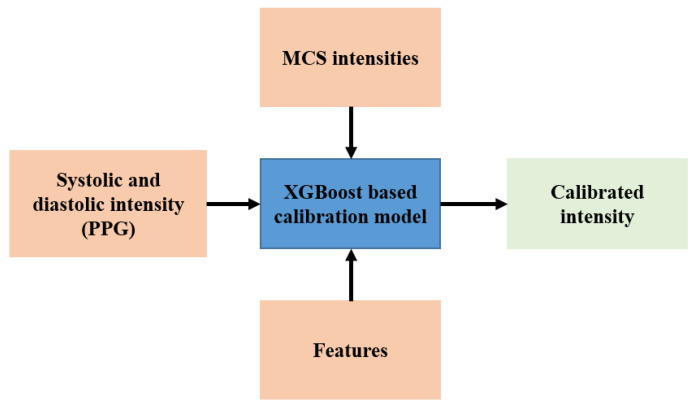
Workflow diagram for calibration.

To calibrate the systolic and diastolic intensities with the MCS intensities, a supervised machine-learning model, XGBoost, was used. The inputs to the calibration model are PPG intensities (systolic and diastolic), skewness and kurtosis of the PPG signals, BMI, and pulse rates of the subjects. The target for the calibration model is the MCS intensity. It is important to note that the calibration was performed separately for systolic and diastolic intensities and no personalized calibration was used in this study. Since the skewness can distinguish the periodic, irregular signals, sudden jumps of the signal and the kurtosis represents whether a signal is peaked or flat [35,36]; these features can be used in the calibration model as input. The block diagram of the calibration model is shown in Figure 8.

## 3. Results

In this study, we evaluated the performance of the proposed model on both publicly available and home-made datasets. We also compared the results obtained from these two datasets. In the public dataset, the PPG signal was collected at a wavelength of 905 nm, and in the self-made dataset, the PPG signal was collected at a wavelength of 940 nm. In the proposed hardware, the IR wavelength is 940 nm, and this device is well established and used in our previous studies [5,43]. It is difficult to change the IR LED in the hardware to match the wavelength of the public dataset. However, the authors of [44] categorized 905 nm and 940 nm into the same IR wavelength group (IR-A); it can be seen that there are no significant differences in the penetration depths in skin tissue between 905 and 940 nm [19]. Furthermore, if there are no significant differences in the penetration depths of the two wavelengths in skin tissue for a given measurement site, the collected PPG signals can be said to be identical based on the PPG signal generation principle. Thus, we compared the estimated results of the two datasets used in our study.

Herein, we present the evaluation of the proposed BP estimation model on both the publicly available and self-made datasets. We also present the fluence rate plots of the MCS in the proposed voxel-based finger model and its calibration accuracy. To evaluate the proposed model, we considered five metrics: mean absolute error (MAE), root mean-squared error (RMSE), standard deviation (SD), Pearson’s correlation coefficient (Pearson’s r), and coefficient of determination (R^2^). To visualize the accuracies of the estimated BP and estimation errors, we also present the error grid analysis (EGA) for MAP [45] and Bland–Altman analyses for the SBP and DBP. There are five regions in the EGA plot: A, B, C, D, and E. The points in region A indicate that there are no clinical differences between the reference and estimated values. Points in region B would probably result in benign findings or no treatments. Points in regions C, D, and E may eventually result in unnecessary treatments with moderate non-life-threatening, severe non-life-threatening, and life-threatening consequences for the patient, respectively. The MAP can be calculated from the SBP and DBP as in Equation (12) [46].

(12) $$\mathrm{MAP}=\mathrm{DBP}+\frac{1}{3}\times \left(\mathrm{SBP}-\mathrm{DBP}\right)$$

### 3.1. MCS and Calibration Accuracy

**Figure 9:**
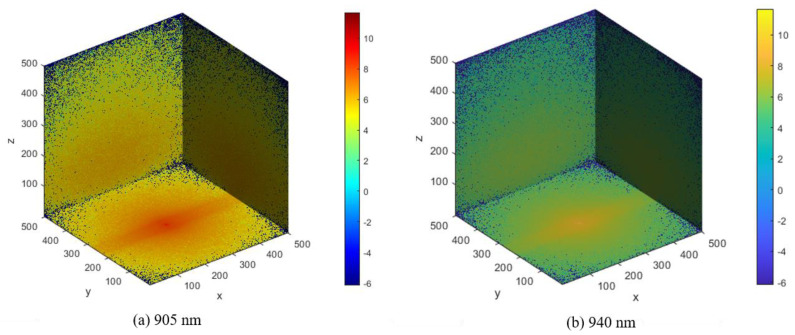
Photon fluence in the voxel-based finger model 3D view.

**Figure 10:**
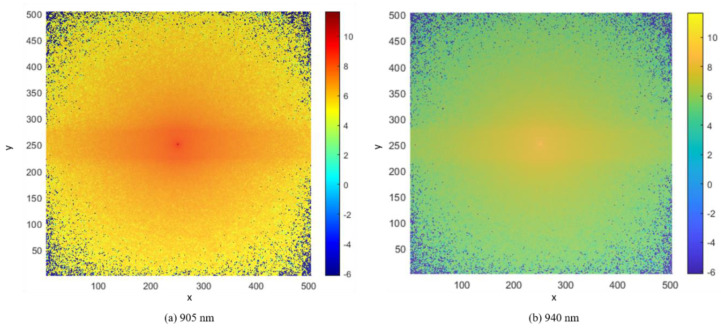
Photon fluence in the voxel-based finger model XY-plane view.

In our study, the voxel-based MCS algorithm was used to propagate photons in the proposed finger model for estimating BP. The Monte Carlo photon propagation was performed on a 10.04 mm thick slab designed for the whole finger. The transmission type used wavelengths of 905 nm and 940 nm, and the reflection type used a wavelength of 940 nm. In the case of reflection-type simulation, the optical source and detector were separated by 0.4 mm, while for the transmission-type simulation, the detector was placed 10.06 mm away from the opposite side of the model. Simulations were performed on a 64-bit operating system (Windows OS) with 20 GB RAM and an Intel Core i7 CPU. The scattering events at the specific wavelengths are depicted by the photon fluence rate plot in a voxel-based finger model. Figure 9 and Figure 10 show the fluence rates throughout the voxel-based finger model in 3D and XY plane views, respectively. The voxel unit (1 unit = 0.02 mm) is represented by the dots on the axes, and the color bars depict the scattering events from minimum to maximum. The maximum number of scattering events was found near the artery, that is, between the upper blood net dermis and reticular dermis, as shown in Figure 9 and Figure 10. Since the diameter of the artery and absorption coefficient change as the BP varies, the maximum scattering events occur near the artery. As previously stated, there are no significant differences between 905 and 940 nm in terms of the penetration depths in skin tissues. At both wavelengths, the maximum scattering events occur near the artery, which is another reason for comparing the evaluation results between 905 and 940 nm.

**Figure 11:**
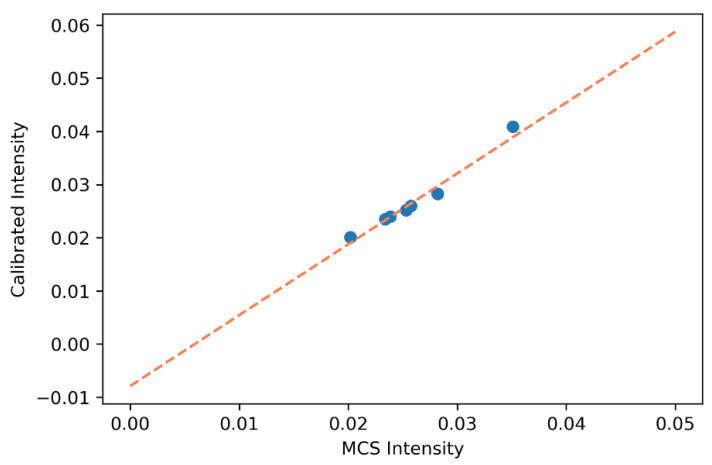
Fitted scatter plot of the calibration of the public dataset and MC photon intensity.

To determine the accuracy of the calibration model (XGBoost), we performed a repeated k-fold cross-validation, which is a technique for improving the estimation performance of machine-learning models. It is also a standard method of estimating model performance. In our study, we used the k-fold cross-validation to test the accuracy of the XGBoost-based calibration model. A 10-fold cross-validation process was repeated three times to estimate the model performance. The average R^2^ value was found to be 0.984 for the public dataset and 0.979 for the self-made dataset. The dataset sizes for training and testing to perform the calibration were 90% and 10%, respectively. Figure 11 shows the fitted curves of the calibration performed on the publicly available dataset and MC photon intensity. The parameters of the XGBoost calibration model and feature importance plots of the input features during calibration are presented in the Supplementary Document (see Section S2 of the supplementary document).

### 3.2. Blood Pressure Estimation (Public Dataset)

**Figure 12:**
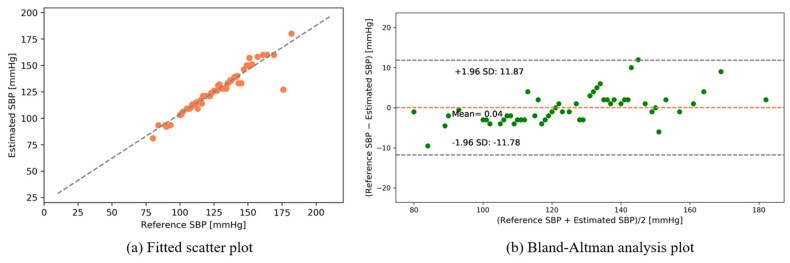
SBP estimation (public dataset).

**Figure 13:**
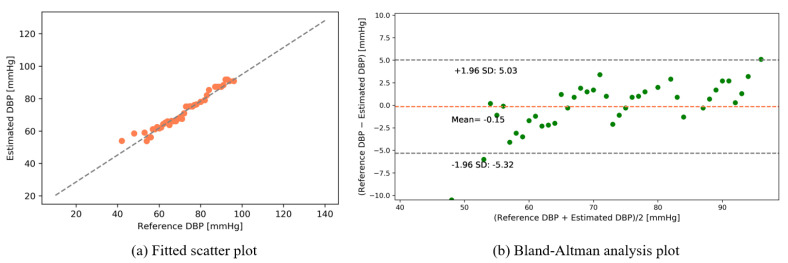
DBP estimation (public dataset).

The following results were obtained after interpolating the calibrated intensity of the PPG signal with the data table of photon intensities (see Table S1 of the Supplementary Document). Figure 12 and Figure 13 show the fitted scatter plots and Bland–Altman analyses of the estimated SBP and DBP, respectively. Figure 12b indicates that the estimated SBP provides a bias of 0.04 ± 6.04 with limits of agreement (95%, 1.96 SD) ranging from −11.78 to 11.87. Figure 13b shows that the DBP estimation provides a bias of −0.15 ± 2.64 with limits of agreement (95%, 1.96 SD) ranging from −5.32 to 5.03.

**Figure 14:**
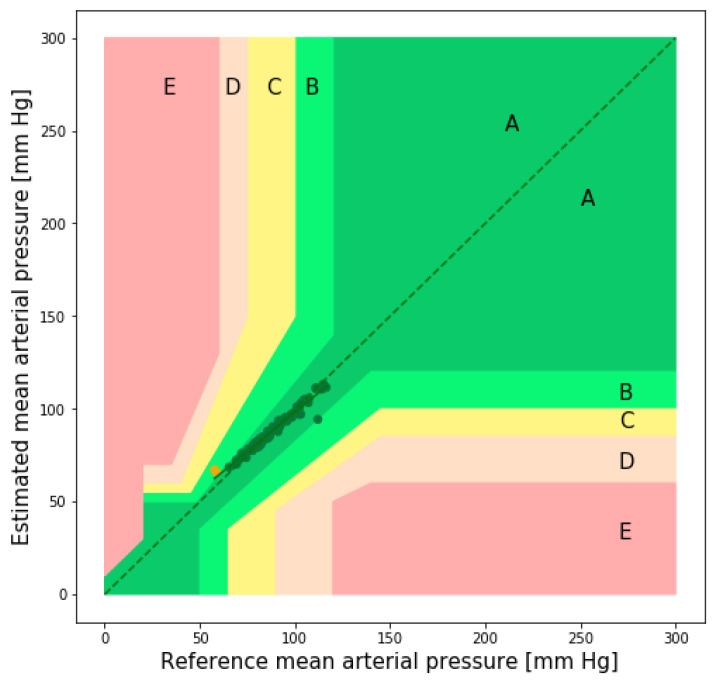
Error grid analysis plot of the MAP (public dataset).

Figure 14 shows the EGA plot of the MAP. As can be seen from the figure, there are a negligible number of points in region B. The values of the evaluation metrics for the SBP, DBP, and MAP estimations are listed in Table 9.

### 3.3. Blood Pressure Estimation (Self-Made Dataset)

#### 3.3.1. Transmission-Type MCS

**Figure 15:**
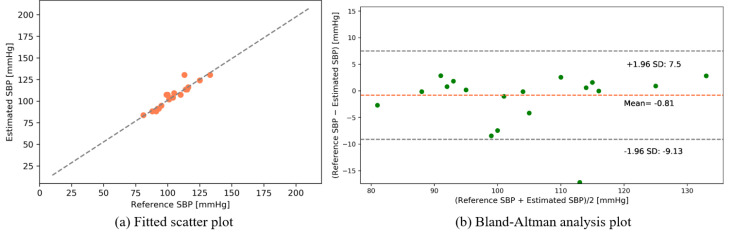
SBP estimation (self-made dataset).

**Figure 16:**
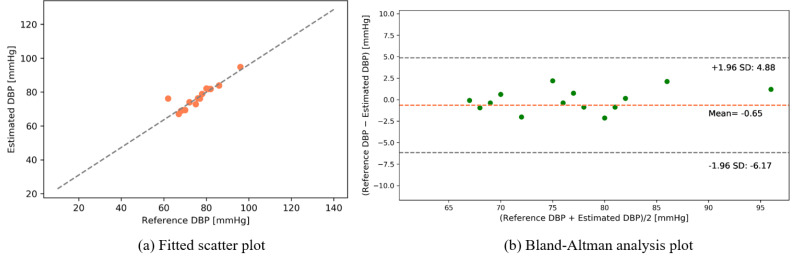
DBP estimation (self-made dataset).

The following results were found from our dataset for the transmission-type MCS. Figure 15 and Figure 16 depict the fitted scatter plots and Bland–Altman analyses of the estimated SBP and DBP, respectively. Figure 15b indicates that the estimated SBP provides a bias of −0.81 ± 4.23 with limits of agreement (95%, 1.96 SD) ranging from −9.13 to 7.5. Figure 16b shows that the DBP estimation provides a bias of −0.65 ± 2.82 with limits of agreement (95%, 1.96 SD) ranging from −6.17 to 4.88.

**Figure 17:**
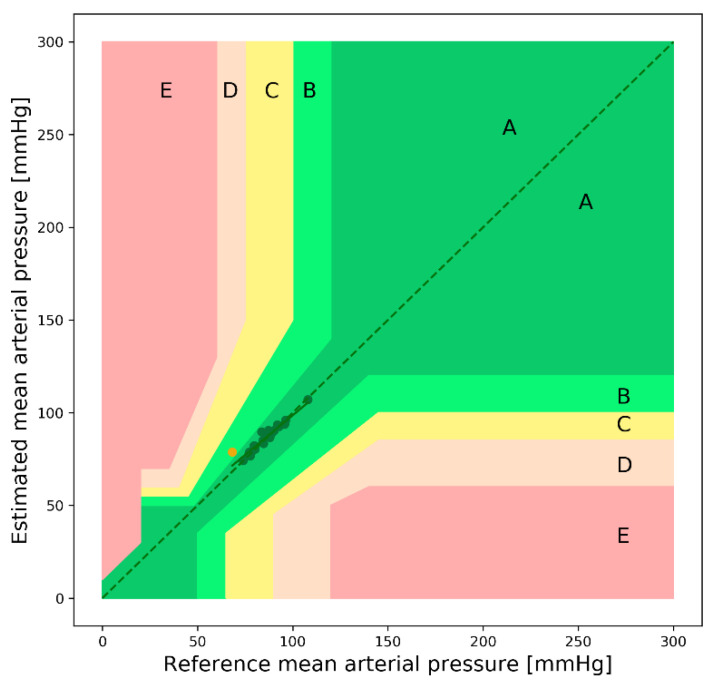
Error grid analysis plot of the MAP (self-made dataset).

In Figure 17, the EGA plot of the MAP is shown, and it is seen from the figure that there are no points in the C, D, and E regions. The values of the evaluation metrics for the SBP, DBP, and MAP estimations are listed in Table 10.

#### 3.3.2. Reflection-Type MCS

**Figure 18:**
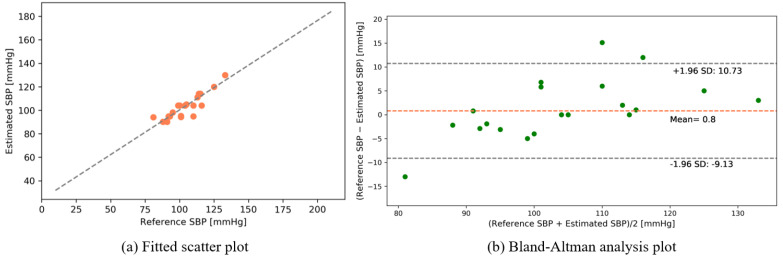
SBP estimation (self-made dataset).

**Figure 19:**
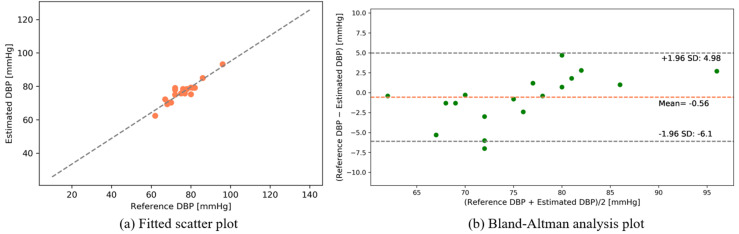
DBP estimation (self-made dataset).

The following results were obtained from our dataset for the reflection-type MCS. Figure 18 and Figure 19 depict the fitted scatter plots and Bland–Altman analyses of the estimated SBP and DBP, respectively. Figure 18b indicates that the estimated SBP provides a bias of 0.8 ± 5.06 with limits of agreement (95%, 1.96 SD) ranging from −9.13 to 10.73. Figure 19b shows that the DBP estimation provides a bias of −0.56 ± 2.82 with limits of agreement (95%, 1.96 SD) ranging from −6.1 to 4.98.

**Figure 20:**
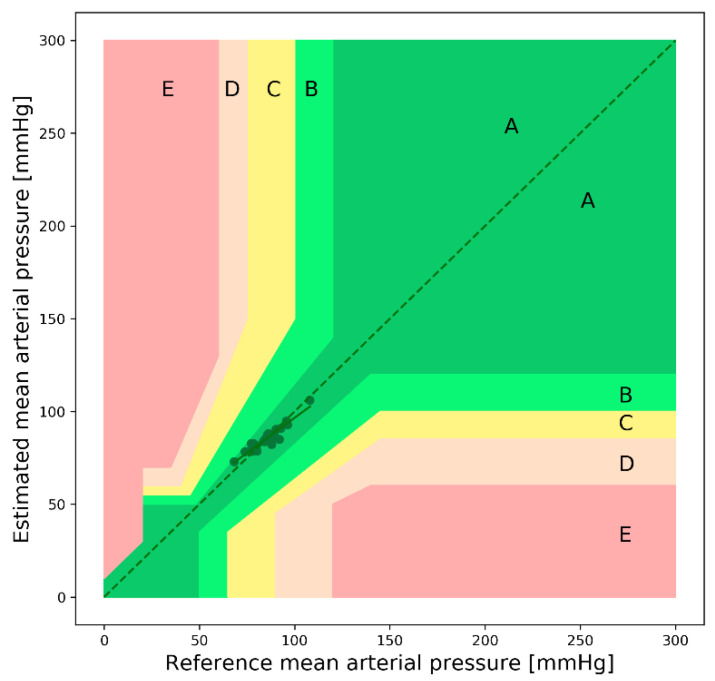
Error grid analysis plot of the MAP (self-made dataset).

In Figure 20, the EGA plot of the MAP is shown; it can be seen from the figure that there are no points in the B, C, D, and E regions. The values of the evaluation metrics for the SBP, DBP, and MAP estimations are listed in Table 11.

### 3.4. Compliance with Standards

The obtained results were compared with the Association for the Advancement of Medical Instrumentation (AAMI) error range. According to the AAMI standard, the mean difference and standard deviation must be less than or equal to 5 ± 8 mmHg [47]. The estimations using our model for both the public and self-made datasets are fully acceptable for the SBP and DBP values. The comparison with the AAMI standard is shown in Table 12. From this table, it is seen that the BP estimation with our model satisfies the AAMI standard as the MAE value is within 5 ± 8 mmHg.

The accuracy of the model was also checked from the point of view of the British Hypertension Society (BHS) grading standard [48]. The BHS grading standard and cumulative error percentage of our data are shown in Table 13. From the results, it is seen that the SBP and DBP estimations using our model fall within “Grade A” for both the public and self-made datasets

We compared the performances of our model for the two datasets. As previously stated, there are no significant differences between the 905 nm and 940 nm wavelengths in terms of PPG signal acquisition, because the penetration depths of these two wavelengths in skin tissue are almost identical. Therefore, we compared the results of the transmission-type MCS between the public and self-made datasets. The MAEs for SBP, DBP, and MAP in the public dataset for the transmission-type MCS are 3.32 ± 6.03 mmHg, 2.02 ± 2.64 mmHg, and 1.76 ± 2.8 mmHg, respectively. The self-made dataset was used for both transmission- and reflection-type MCS. In the transmission mode, the MAEs are 2.54 ± 4.24 mmHg for SBP, 1.49 ± 2.82 mmHg for DBP, and 1.51 ± 2.41 mmHg for MAP. In the reflection type, the MAEs are 3.35 ± 5.06 mmHg for SBP, 2.07 ± 2.83 mmHg for DBP, and 2.12 ± 2.83 mmHg for MAP. It can be seen that in the transmission type for the self-made dataset, the estimation results have less errors than the reflection type for the self-made dataset; this is because the performance of the estimation model depends on the MCS, so more accurate photon intensities are recorded during the transmission-type MCS. The artery is considered to be located between the upper blood net dermis and reticular dermis in our proposed voxel-based MCS model, so that while propagating photons into the model, the photodetector can record the intensities of the photons transmitted through the finger model. These photon intensities are more accurate than those recorded during the reflection type. With relatively more accurate photon intensities, the XGBoost-based calibration model performs well on the systolic and diastolic intensities of the PPG signals, thus resulting in slightly better performance.

### 3.5. Comparisons with Related Works

In general, similar studies in this field are difficult to evaluate owing to various evaluation criteria and insufficient datasets. However, we compared the results of our proposed model with previous works that have used PPG signals for estimating the SBP and DBP. For comparing performances, we considered the MAE, SD, and Pearson’s r of the estimation models. Table 14 shows the performance comparisons of these studies. In the table, the performance evaluation metrics are listed for both datasets used in our model. The Pearson’s r values for the SBP, DBP, and MAP estimations also prove the estimation accuracy of our model. Although the Pearson’s r value is slightly lower for the self-made dataset, it satisfies the AAMI and BHS standards with small MAE and SD values.

While reviewing other relevant studies, Chowdhury et al. [7] used a dataset similar to that used to validate our model for the public dataset; they used a Gaussian process regression (GPR) to estimate the SBP and DBP. They also used feature selection techniques in their study to provide inputs to machine-learning models. However, in our study, only four features were used to calibrate the model and obtain lower errors. Furthermore, as shown in Table 10 and Table 12, SBP estimations with their approach do not meet the AAMI requirements. In [8], the authors proposed a U-net deep-learning architecture to estimate the ABP waveforms and calculate the SBP, DBP, and MAP from these waveforms. Since the deep-learning-based model has to estimate the ABP waveforms for estimating BP, it can provide erroneous results when there is a lack of stable PPG signals. In Table 14, it is seen that our proposed model outperforms the model of [8] in terms of MAE and SD values, which are important parameters for validating the estimation results compared to the AAMI and BHS standards. Xie et al. [17] used the random forest model to estimate the SBP and DBP. In terms of the MAE and SD, our proposed model outperforms the random forest model on both the public and self-made datasets. Furthermore, our proposed model outperforms the model in [16] for all evaluation parameters.

Compared with the above models, our MCS-based model obtains lower MAE and SD, with relatively similar accuracy for estimating the SBP and DBP values. Therefore, our proposed model can be said to be more accurate for estimating SBP and DBP according to the AAMI and BHS standards.

## 4. Conclusions

Cuffless and comfortable BP measurements are currently an active area of research. In this study, we proposed a cuffless BP estimation model based on MCS. After MCS for a specific range of blood pressure, photon intensities were recorded to generate a data table. PPG signals from both a publicly available dataset (number of subjects = 100) and self-made dataset (number of subjects = 30) have been used to evaluate the proposed model. The systolic and diastolic photon intensities detected in real PPG signals were calibrated using a supervised machine learning based calibration model. Both transmission- and reflection-type MCSs were performed for estimating the SBP and DBP, from which we also calculated the MAP. We present the EGA plots for MAP and Bland–Altman analyses for the SBP and DBP to evaluate our model performance. The EGA plot for MAP proves the accuracy of our estimations for use in regular BP measurements, and the Bland–Altman analysis shows the errors of the SBP and DBP estimation results. From the Bland–Altman plot, it can also be concluded that the performance of the proposed model is within the margin of error, as most of the estimated values are within ±1.96 SD. Therefore, our proposed model satisfies the standards of the AAMI and complies with the ‘Grade A’ category according to the BHS.

Previous studies in the field of blood pressure (BP) estimation [7,8,16,17] focused on using machine learning or deep learning models for BP estimation, which often create obstacles in estimating the desired parameters accurately. Therefore, to reduce the possibility of inaccurate estimation of BP, we have proposed a finger model to execute MCS for recording the precise intensities during the systolic and diastolic phases of the PPG signal. In addition, in this study, a relationship between BP and artery diameter is also deduced. The heterogeneous finger model proposed in this study provides a more precise design of the model and more accurate results within the range that can provide a chance to optimize the model. Since earlier studies in this field focused on machine or deep learning models for BP estimation, there is little scope for optimizing the model as well as model complexity.

In our study, the thickness of the finger model is considered as a constant for all simulations but may vary from person to person in real scenarios. Therefore, to evaluate the model performance more accurately, it will be necessary to create a self-made dataset from more subjects. Despite the limitations of the estimation model, the proposed method can be used for BP monitoring without the use of a cuff. We expect that our proposed model will provide an easy cuffless BP estimation method to help people plan healthy lifestyles.

## Figures and Tables

**Table 1:** Blood pressure ranges for various health conditions.

Blood Pressure Category	SBP [mmHg]	DBP [mmHg]
Normal	Less than 120	Less than 80
Elevated	120–129	Less than 80
Hypertension stage 1	130–139	80–89
Hypertension stage 2	140 or higher	90 or higher
Hypertensive crisis	Higher than 180	Higher than 120

**Table 2:** Thicknesses of the layers of the proposed finger model [9,20,21,22].

Layer	Thickness [mm]
Stratum corneum	0.02
Epidermis	0.25
Papillary dermis	0.1
Upper blood net dermis	0.08
Reticular dermis	0.2
Deep blood net dermis	0.3
Fat	0.55
Muscle	1.5
Bone	2

**Table 3:** Blood and water volume fractions used in [23].

Layer	Blood Volume Fraction, $V_{b}\;(\%)$	Water Volume Fraction, $V_{wat}\;(\%)$
Stratum corneum	0	5
Epidermis	0	20
Papillary dermis	5	50
Upper blood net dermis	20	60
Reticular dermis	4	70
Deep blood net dermis	10	70
Fat	0	70
Muscle	0	70
Bone	0	0

**Table 4:** Bio-optical properties of the proposed finger layers.

Layers	$\mathrm{Absorption}\;\mathrm{Coefficient},\;\mu_{a}$ [mm^−1^]		$\mathrm{Scattering}\;\mathrm{Coefficient},\;\mu_{s}$ [mm^−1^]		Anisotropy, *g*	Refractive Index, *n*
	905 nm	940 nm	905 nm	940 nm		
Stratum corneum	0.11350	0.09745	100		0.86	1.5
Epidermis	0.16825	0.21397	45		0.8	1.34
Papillary dermis	0.09631	0.23308	30		0.9	1.4
Upper blood net dermis	0.09864	0.24202	35		0.95	1.39
Reticular dermis	0.09824	0.29906	25		0.8	1.4
Deep blood net dermis	0.10118	0.32549	30		0.95	1.38
Fat	0.0142	0.0170	6.33	5.42	0.8	1.37
Muscle	0.031	0.0401	1.83	5.81	0.5	1.37
Bone	0.15	0.0457	15.2	24.70	0.92	1.37
Artery	-	-	4.85		0.8	1.39

**Table 5:** Absorption coefficients of whole blood and vessel wall.

$\mu_{blood}\;{\mathrm{mm}}^{-1}\;(\mathrm{Whole}\;\mathrm{Blood}$ [26])		$\mu_{vw}\;{\mathrm{mm}}^{-1}\;(\mathrm{Vessel}\;\mathrm{Wall}$ [27])	
905 nm	940 nm	905 nm	940 nm
0.011160	0.011778	0.014869	0.009316

**Table 6:** Statistical information of the dataset.

	SBP [mmHg]	DBP [mmHg]	BMI	Pulse Rate	Age [Years]
Min	80	42	14.69	52	21
Max	182	107	37.46	106	86
Mean	127.9	71.9	23.11	73.64	57.2
SD	20.33	11.09	3.99	10.71	15.84

Min: minimum, Max: maximum, SD: standard deviation.

**Table 7:** Statistical information of the selected subjects.

	SBP [mmHg]	DBP [mmHg]	BMI	Pulse Rate	Age [Years]
Min	80	42	15.94	52	23
Max	182	96	35.84	103	85
Mean	124.42	69.46	23.23	73.2	54.7
SD	19.65	10.46	3.88	10.87	16.35

**Table 8:** Statistical information from the subjects (No. of subjects = 30).

	SBP [mmHg]	DBP [mmHg]	BMI	Pulse Rate	Age [Years]
Min	81	62	20.76	55	25
Max	133	96	34.6	108	61
Mean	104.57	75.8	27.87	82.47	30.4
SD	11.65	6.86	2.96	10.49	7.62

**Table 9:** Evaluation metrics (public dataset).

Metrics	MAE [mmHg]	RMSE [mmHg]	SD [mmHg]	Pearson’s r	R^2^
SBP	3.32	6.01	6.03	0.95	0.91
DBP	2.02	2.65	2.64	0.97	0.94
MAP	1.76	2.83	2.8	0.98	0.94

**Table 10:** Evaluation metrics for SBP, DBP, and MAP (self-made dataset).

Metrics	MAE [mmHg]	RMSE [mmHg]	SD [mmHg]	Pearson’s r	R^2^
SBP	2.54	4.31	4.24	0.94	0.86
DBP	1.49	2.89	2.82	0.91	0.82
MAP	1.51	2.52	2.41	0.95	0.90

**Table 11:** Evaluation metrics for SBP, DBP, and MAP.

Metrics	MAE [mmHg]	RMSE [mmHg]	SD [mmHg]	Pearson’s r	R^2^
SBP	3.35	5.12	5.06	0.90	0.81
DBP	2.07	2.88	2.83	0.91	0.82
MAP	2.12	2.83	2.83	0.95	0.87

**Table 12:** Comparison of our results with the AAMI standard.

		No. of Subjects	MAE [mmHg]	SD [mmHg]
AAMI [47]		>85	≤5	≤8
Public dataset	SBP	100	3.32	6.03
	DBP		2.02	2.64
Self-made dataset (Transmission)	SBP	30	2.54	4.24
	DBP		1.49	2.81
Self-made dataset (Reflection)	SBP	30	3.35	5.06
	DBP		2.07	2.83

**Table 13:** Comparisons of the results with the BHS grading standard.

		Cumulative Error (%)		
BHS grading standard [48]		≤5 mmHg	≤10 mmHg	≤15 mmHg
	Grade A	60%	85%	95%
	Grade B	50%	75%	90%
	Grade C	40%	65%	85%
Public dataset	SBP	91%	98%	98%
	DBP	96%	96%	99%
Self-made dataset (Transmission)	SBP	86.67%	96.67%	96.67%
	DBP	96.67%	96.67%	99%
Self-made dataset (Reflection)	SBP	80%	90%	96.67%
	DBP	86.67%	96.67%	98%

**Table 14:** Performance comparisons with related works.

Model	Dataset	SBP/DBP/MAP			
		MAE [mmHg]	SD [mmHg]	RMSE [mmHg]	Pearson’s r
Proposed (Public dataset)	Public dataset [33]	3.32/2.02/1.76	6.03/2.64/2.8	6.01/**2.65**/2.83	0.95/**0.97/0.98**
Proposed (self-made)	Transmission	**2.54**/**1.49/1.51**	**4.24**/2.81/**2.41**	**4.31**/2.89/**2.52**	0.94/0.91/0.95
	Reflection	3.35/2.07/2.12	5.83/**2.06**/2.83	5.12/2.88/2.83	0.90/0.91/0.95
Chowdhury et al. [7]	Public dataset [33]	3.02/1.74/-	9.29/5.54/-	6.74/3.59/-	0.95/0.96/-
Athaya et al. [8]	MIMIC II and MIMIC III	3.68/1.97/2.17	4.42/2.92/3.06	5.75/3.52/3.75	**0.976/0.970/**0.976
Esmaelpoor et al. [16]	MIMIC II	4.21/3.24/-	7.59/5.39/-	7.57/5.40/-	0.938/0.942/-
Xie et al. [17]	Queensland Vital Signs Dataset	3.97/2.10/-	5.55/2.84/-	-	0.95/0.95/-

Best values are in boldface font.

## Data Availability

We have created our own dataset for this study. Since further re-search are on processing, we cannot publish the dataset right now.

## Supplementary Materials

The following supporting information can be downloaded at: https://www.mdpi.com/article/10.3390/s22031175/s1, Table S1: MCS intensities and the blood pressure (BP); Figure S1: Feature importance plots in calibration of public dataset; Figure S2: Feature importance plots in calibration of self-made dataset; Table S2: Calibration accuracy parameters; Figure S3: Fitted scatter plot of calibration (Public Dataset); Figure S4: Fitted scatter plot of calibration (Self-made Dataset, SBP); Figure S5: Fitted scatter plot of calibration (Self-made Dataset, SBP); Table S3: Data information of self-made dataset.
