# Supplementary material for: Cuffless Blood Pressure Estimation Based on Monte Carlo Simulation Using Photoplethysmography Signals
Source: Sensors (Basel). 2022 Feb 4;22(3):1175. doi: 10.3390/s22031175 (PMC8838459; doi:10.3390/s22031175)
Supplement: Supplementary file 1 [file sensors-22-01175-s001.zip › sensors-1563223-supplementary.pdf]

Supplementary Document

Cuffless Blood Pressure Estimation Based on Monte Carlo Simulation Using PPG Signals

Chowdhury Azimul Haque, Tae-Ho Kwon, and Ki-Doo Kim*
Department of Electronics Engineering, Kookmin University, Seoul 02707, Korea
Correspondence: kdk@kookmin.ac.kr

S1. Monte Carlo Simulation (MCS) photon intensity

Monte Carlo Simulation (MCS) photon intensities are listed for the blood pressure (BP) range defined in the manuscript.

Table S1. MCS intensities and the blood pressure (BP)

BP [mmHg]	MCS Intensities		
	905 nm [Transmission]	940 nm [Transmission]	940 nm [Reflection]
42	0.02711784	0.020072	0.8457336
43	0.02711812	0.020076	0.8457376
44	0.02711984	0.02007818	0.8457416
45	0.02711902	0.02007964	0.8457584
46	0.03101134	0.02946005	0.8484837
47	0.02795807	0.0426676	0.8441908
48	0.02010229	0.03671348	0.8462939
49	0.02010178	0.03671433	0.8463086
50	0.02361568	0.04363214	0.8396249
51	0.0255546	0.0280922	0.8428138
52	0.02373627	0.03933859	0.8455392
53	0.02345043	0.02783998	0.8457364
54	0.02344988	0.02784039	0.8457496
55	0.02270353	0.03082046	0.846635
56	0.02236041	0.0377798	0.8469514
57	0.02387042	0.02582704	0.8489993
58	0.02399026	0.04341904	0.8488946
59	0.01977873	0.04653006	0.8464148
60	0.04086005	0.03523245	0.8494492
61	0.02608989	0.05229087	0.849858
62	0.02826967	0.04176609	0.8466956
63	0.02602045	0.03717267	0.8451415
64	0.02107668	0.04021375	0.8439127
65	0.02295449	0.03919424	0.8474678
66	0.0282323	0.03613778	0.8481388

67	0.0236647	0.03820538	0.8505212
68	0.02255525	0.04003597	0.851362
69	0.02009885	0.04058646	0.8537383
70	0.02009859	0.04058692	0.8537477
71	0.02230179	0.03824981	0.850168
72	0.02114777	0.03536275	0.8500022
73	0.01567085	0.03546827	0.8491877
74	0.01567056	0.03546872	0.8491962
75	0.02831517	0.03891068	0.8479552
76	0.03184482	0.04399347	0.8492959
77	0.02949651	0.04250846	0.8481393
78	0.0290099	0.05527233	0.8488091
79	0.02784513	0.03236006	0.8511328
80	0.02784469	0.03236037	0.8511406
81	0.02395297	0.03598937	0.8558292
82	0.0239527	0.03598973	0.8558365
83	0.02395244	0.03599009	0.8558438
84	0.02524699	0.03744824	0.8513798
85	0.02702625	0.03961039	0.8500157
86	0.02567553	0.03461762	0.8497519
87	0.02522651	0.05494636	0.8506472
88	0.02522627	0.05494687	0.8506536
89	0.02522603	0.05494736	0.8506602
90	0.0252258	0.05494785	0.8506665
91	0.02522557	0.05494833	0.8506731
92	0.01918853	0.05546023	0.8501546
93	0.01918833	0.05546065	0.8501611
94	0.02317976	0.03975791	0.8475047
95	0.0354873	0.04631997	0.8523362
96	0.03548685	0.04632032	0.8523423
97	0.02700135	0.05169496	0.8505083
98	0.03318263	0.03676847	0.8522965
99	0.03318233	0.03676873	0.8523023
100	0.03318203	0.03676898	0.8523079
101	0.02669386	0.03767198	0.8509209
102	0.03023727	0.03838144	0.8535916
103	0.034879	0.0434528	0.8560116
104	0.03487864	0.0434532	0.856017
105	0.03636313	0.03208868	0.8525602
106	0.03636279	0.0320889	0.8525652
107	0.03636245	0.03208912	0.8525706

108	0.04451417	0.03953046	0.8551561
109	0.03005495	0.03702199	0.8501392
110	0.03005469	0.03702219	0.8501443
111	0.03071236	0.04314275	0.8514249
112	0.04423176	0.03773462	0.8495333
113	0.02770238	0.04023519	0.8485356
114	0.03369722	0.03311976	0.8520494
115	0.03369692	0.03312007	0.8520537
116	0.03255456	0.0293572	0.8564828
117	0.02340873	0.0435042	0.8560033
118	0.02344839	0.0491522	0.8548808
119	0.0234482	0.04915252	0.8548854
120	0.023448	0.04915283	0.85489
121	0.02344782	0.04915313	0.8548943
122	0.02347782	0.03289374	0.8520371
123	0.03234539	0.04845358	0.853301
124	0.03234514	0.04845388	0.8533052
125	0.02547963	0.01643657	0.8559254
126	0.02547941	0.01643666	0.8559297
127	0.02547919	0.01643675	0.8559335
128	0.01240404	0.0202831	0.857021
129	0.02423909	0.03137306	0.855604
130	0.02848372	0.03993193	0.8590465
131	0.02848352	0.03993217	0.8590503
132	0.02848334	0.0399324	0.8590544
133	0.02848315	0.03993263	0.8590582
134	0.02848296	0.03993286	0.8590621
135	0.03044824	0.02788507	0.858614
136	0.02599764	0.03589552	0.8587447
137	0.01418257	0.04978543	0.8574721
138	0.01418243	0.04978566	0.8574756
139	0.02411954	0.05549416	0.8572748
140	0.02392396	0.04413134	0.85667
141	0.02392378	0.04413163	0.8566738
142	0.02666388	0.0374083	0.8559338
143	0.0300696	0.04981466	0.8540357
144	0.03006943	0.04981492	0.8540391
145	0.03006925	0.04981518	0.8540424
146	0.03006909	0.04981543	0.854046
147	0.0314962	0.04329025	0.8529058
148	0.03433188	0.04298313	0.8561689

149	0.0344267	0.04133921	0.8570251
150	0.0344265	0.04133938	0.8570284
151	0.03442632	0.04133953	0.8570316
152	0.03442613	0.04133968	0.8570347
153	0.03241206	0.05031306	0.8579315
154	0.0254697	0.03691802	0.8534366
155	0.02546954	0.03691816	0.85344
156	0.02546938	0.0369183	0.8534429
157	0.02546922	0.03691845	0.8534462
158	0.02546906	0.03691859	0.8534495
159	0.02546891	0.03691872	0.8534526
160	0.02546876	0.03691886	0.8534555
161	0.02584452	0.05057871	0.8560205
162	0.02584439	0.0505789	0.8560235
163	0.02584427	0.05057908	0.8560266
164	0.02959988	0.0504461	0.8583384
165	0.03481115	0.05373619	0.8533676
166	0.03481099	0.05373638	0.8533705
167	0.03481084	0.05373658	0.8533732
168	0.03481069	0.05373676	0.8533762
169	0.03481054	0.05373695	0.8533791
170	0.03310201	0.04868141	0.8545665
171	0.03310174	0.04868155	0.8545693
172	0.03310149	0.04868169	0.8545721
173	0.04795573	0.0370426	0.8567592
174	0.04795552	0.03704274	0.8567621
175	0.04795533	0.03704289	0.8567645
176	0.03407685	0.04573769	0.8566774
177	0.03407666	0.04573789	0.8566796
178	0.03407646	0.04573808	0.8566825
179	0.03407627	0.04573828	0.8566851
180	0.03188191	0.04900373	0.855634
181	0.04101169	0.04553554	0.857867
182	0.04101152	0.04553569	0.8578697
183	0.0313578	0.03298684	0.8559335
184	0.02743777	0.04878032	0.8570455
185	0.02743764	0.04878048	0.8570482
186	0.02743752	0.04878063	0.8570505
187	0.02743739	0.0487808	0.8570531
188	0.02743727	0.04878095	0.8570555
189	0.02347308	0.03600044	0.8557512

190	0.0278311	0.03458031	0.8558176
191	0.02783093	0.03458041	0.8558199
192	0.02783077	0.03458051	0.8558221
193	0.03094488	0.04665593	0.8572981
194	0.02756397	0.02151029	0.8553388
195	0.02756383	0.02151039	0.8553411
196	0.0275637	0.02151049	0.8553436
197	0.02756356	0.02151058	0.855346
198	0.02756343	0.02151068	0.8553483
199	0.0275633	0.02151078	0.8553504
200	0.02756317	0.02151087	0.8553529

S2. Intensity calibration feature importance and accuracy

For the XGBoost based calibration model, the default parameters were used. The values of the most common model parameters are given as follows:

i) Learning rate: 0.3
ii) Maximum depth: 6
iii) Number of estimators: 100
iv) Objective function: Squared Error (reg:suqarederror)

The feature importance plots for different input features in calibration are shown in Figures S1 and S2.

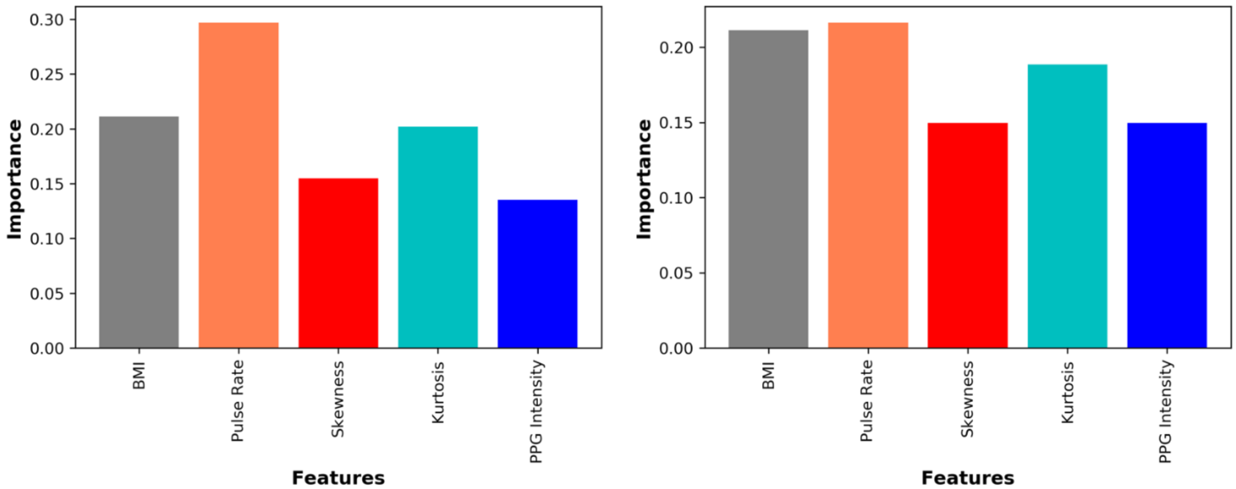

(a) SBP calibration (a) DBP calibration

Figure S1. Feature importance plots in calibration of public dataset.

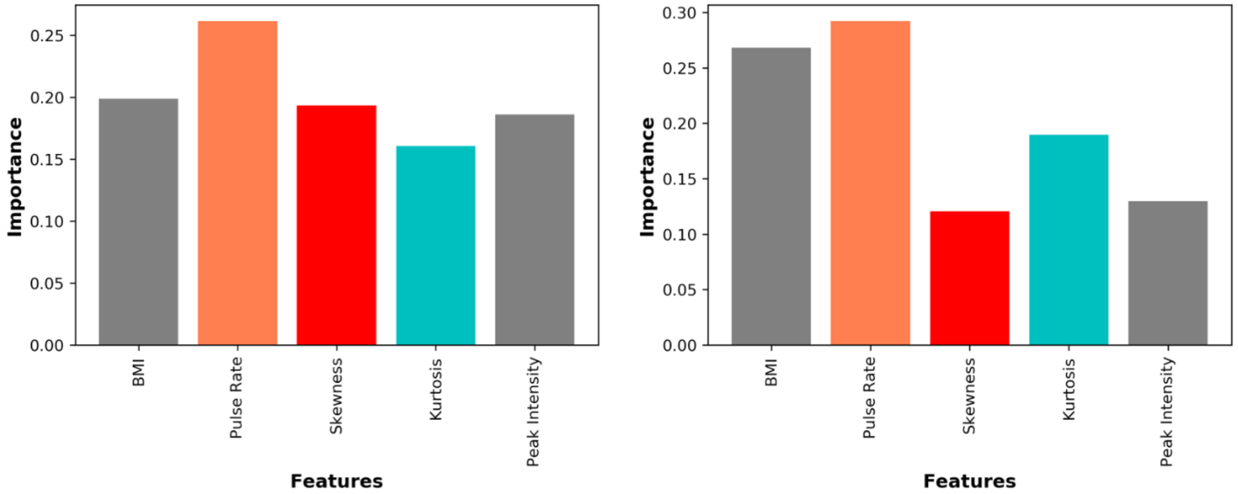

(a) SBP calibration (b) DBP calibration

Figure S2. Feature importance plots in calibration of self-made

dataset. For accuracy validation:

Table S2. Calibration accuracy parameters

Dataset	Train size	Test size	Average R^2
Public	90% (90 data point)	10% (10 data point)	0.984
Self-made	90% (27 data point)	10% (3 data point)	0.979

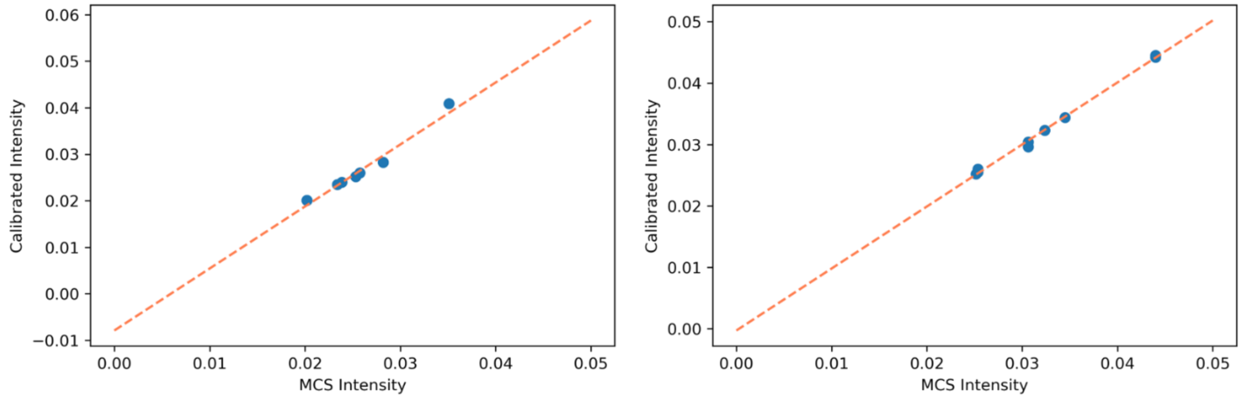

(a) SBP (b) DBP

Figure S3. Fitted scatter plot of calibration (Public Dataset).

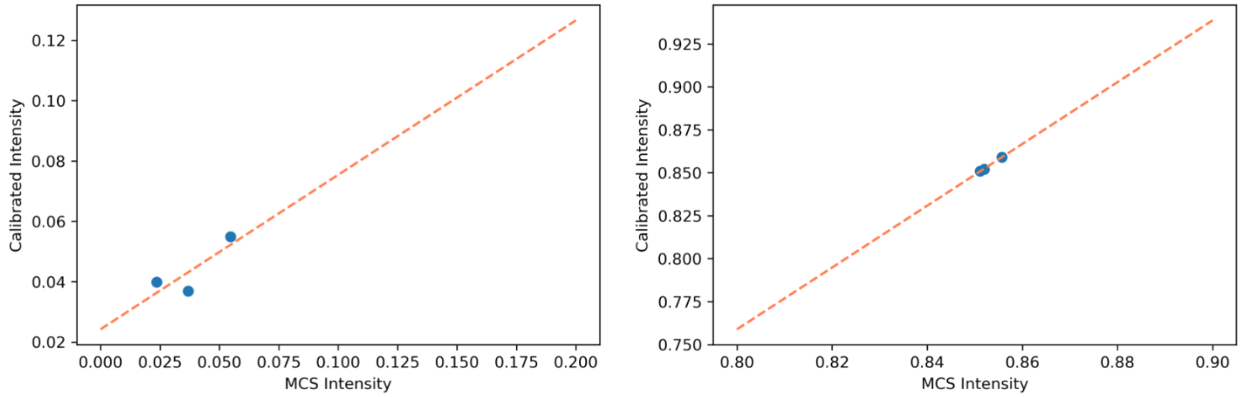

(a) Transmission (b) Reflection

Figure S4. Fitted scatter plot of calibration (Self-made Dataset, SBP).

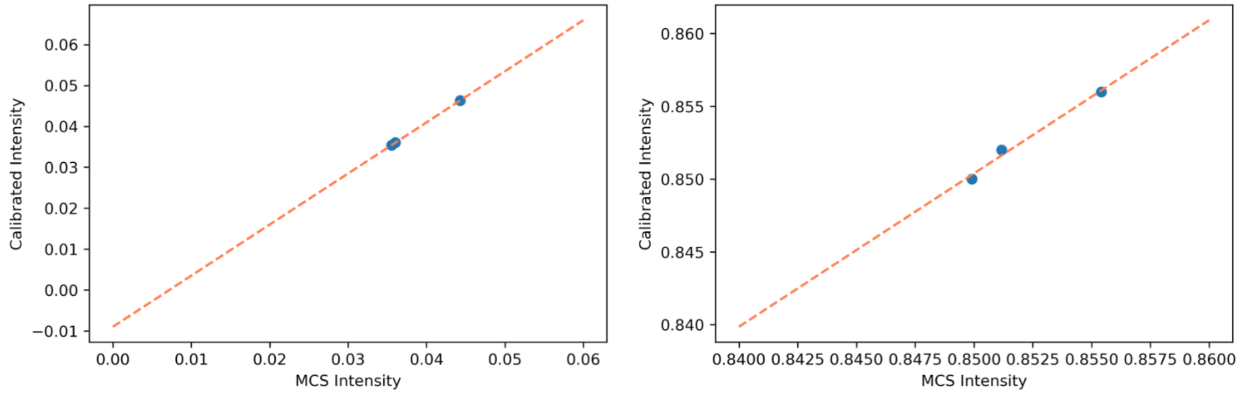

(a) Transmission (b) Reflection

Figure S5. Fitted scatter plot of calibration (Self-made Dataset, DBP).

S3. Self-made dataset information

Table S3. Data information of self-made dataset.

Subject ID	Reference SBP [mmHg]	Reference DBP [mmHg]	Pulse rate	BMI	Height [m]	Weight [kg]	Age [years]	Gender
1	115	78	83	33.74	1.87	118	29	M
2	115	86	79	29.13	1.75	89.2	28	M
3	100	81	97	27.76	1.75	85	32	M
4	91	72	84	26.04	1.58	65	25	F
5	100	70	75	25.06	1.55	60.2	26	F
6	110	77	77	26.61	1.61	69	29	F
7	104	80	88	27.75	1.75	85	32	M
8	92	70	94	29.12	1.75	89.2	28	M
9	81	62	85	25.05	1.55	60.2	26	F

10	104	78	79	26.61	1.61	69	29	F
11	104	82	99	26.03	1.58	65	25	F
12	99	70	67	26.54	1.64	71.4	28	F
13	91	72	80	27.75	1.75	85	32	M
14	133	96	85	26.36	1.72	78	54	M
15	125	82	55	20.76	1.7	60	61	M
16	105	76	102	27.75	1.75	85	32	M
17	104	75	74	25.30	1.8	82	26	M
18	113	69	86	29.12	1.75	89.2	28	M
19	101	70	82	26.03	1.58	65	26	F
20	93	70	78	28.72	1.55	69	29	F
21	88	67	76	29.70	1.61	77	29	F
22	114	82	77	34.68	1.87	120	29	M
23	116	80	79	26.01	1.86	90	31	M
24	95	68	70	27.63	1.58	69	29	F
25	110	77	85	27.75	1.75	85	32	M
26	115	86	92	26.03	1.58	65	25	F
27	101	76	108	27.45	1.65	77	29	F
28	115	78	83	33.74	1.87	118	29	M
29	88	72	76	26.03	1.58	65	26	F
30	115	72	79	29.38	1.75	90	27	M
